# Global trends and regional differences in the burden of anxiety disorders and major depressive disorder attributed to bullying victimisation in 204 countries and territories, 1999–2019: an analysis of the Global Burden of Disease Study

**DOI:** 10.1017/S2045796022000683

**Published:** 2022-11-28

**Authors:** C. Hong, Z. Liu, L. Gao, Y. Jin, J. Shi, R. Liang, M. Maimaitiming, X. Ning, Y. Luo

**Affiliations:** 1 Department of Global Health, School of Public Health, Peking University, Beijing, China; 2 Institute for Global Health and Development, Peking University, Beijing, China; 3 Peking University Sixth Hospital (Institute of Mental Health), National Clinical Research Center for Mental Disorders & Key Laboratory of Mental Health, Ministry of Health (Peking University), Beijing, China; 4 Institute for International and Areas Studies, Tsinghua University, Beijing, China; 5 Department of Epidemiology and Population Health, Stanford University School of Medicine, Stanford, USA; 6 Kunming Medical University, Kunming Yunnan Province, China

**Keywords:** Common mental disorders, depression, mental health, risk factors, stressful life events

## Abstract

**Aim:**

This study aimed to analyse the temporal and spatial trends in the burden of anxiety disorders and major depressive disorder related to bullying victimisation on global, regional and country scales.

**Methods:**

Data were from the 2019 Global Burden of Disease (GBD) Study. We assessed the global disability-adjusted life years (DALYs, per 100 000 population) of anxiety disorders and major depressive disorder attributable to bullying victimisation by age, sex and geographical location. The percentage changes in age-standardised rates of DALYs were used to quantify temporal trends, and the annual rate changes across 204 countries and territories were used to present spatial trends. Furthermore, we examined the relationship between the sociodemographic index (SDI) and the burden of anxiety disorders as well as major depressive disorder attributable to bullying victimisation and its spatial and temporal characteristics globally.

**Results:**

From 1990 to 2019, the global DALY rates of anxiety disorders and major depressive disorder attributable to bullying victimisation increased by 23.31 and 26.60%, respectively, with 27.27 and 29.07% for females and 18.88 and 23.84% for males. Across the 21 GBD regions, the highest age-standardised rates of bullying victimisation-related DALYs for anxiety disorders were in North Africa and the Middle East and for major depressive disorder in High-income North America. From 1990 to 2019, the region with the largest percentage increase in the rates of DALYs was High-income North America (54.66% for anxiety disorders and 105.88% for major depressive disorder), whereas the region with the slowest growth rate or largest percentage decline was East Asia (1.71% for anxiety disorders and −25.37% for major depressive disorder). In terms of SDI, this study found overall upward trends of bullying-related mental disorders in areas regardless of the SDI levels, although there were temporary downward trends in some stages of certain areas.

**Conclusions:**

The number and rates of DALYs of anxiety disorders and major depressive disorder attributable to bullying victimisation increased from 1990 to 2019. Effective strategies to eliminate bullying victimisation in children and adolescents are needed to reduce the burden of anxiety disorders and major depressive disorder. Considering the large variations in the burden by SDI and geographic location, future protective actions should be developed based on the specific cultural contexts, development status and regional characteristics of each country.

## Introduction

Mental disorders are recognised as the leading causes of disease burden, with 1566.2 disability-adjusted life years (DALYs) per 100 000 population globally in 2019 (GBD 2019 Mental Disorders Collaborators, 2022). Of these, depressive disorders (major depressive disorder and dysthymia) accounted for the largest proportion of mental disorder DALYs (37.3%), followed by anxiety disorders (22.9%). The burden of mental disorders was present across the entire lifespan, with the number of DALYs increasing during childhood and adolescence, peaking between 25 and 34 years, and decreasing after age 35 years (GBD 2019 Mental Disorders Collaborators, 2022). Overall, DALY rates due to mental disorders exhibit great regional variations; Western Europe, Sub-Saharan Africa, North Africa and the Middle East were found to have the highest rates, whereas Southeast Asia, East Asia, Central Asia and High-income Asia Pacific had the lowest rates (GBD 2019 Mental Disorders Collaborators, 2022). Life stressors and experiences have been identified as the major causes of mental disorders (UNESCO, 2019). As one of the pervasive stressors, bullying victimisation was reported to be significantly associated with mental disorders, especially increasing the risk of depression and anxiety (CDC, 2020).

Bullying victimisation is defined as the intentional, repeated and negative behaviour by one or more persons directed against a person who has difficulty defending himself or herself (Awiria *et al*., 1994). In addition to physical, verbal, emotional and relational bullying, bullying can also take place over the internet via cyberbullying (Kim *et al*., 2022a, 2022b). Bullying is a serious sociodevelopmental issue globally associated with a range of short- and long-term problems among victims (Maynard *et al*., 2016). According to one UNESCO report, almost one-third of students were bullied by peers at school at least once in the last month (UNESCO, 2019). While a higher global prevalence of bullying occurs among boys (34.8%) than among girls (30.4%) (He *et al*., 2022), girls may suffer more psychosomatic effects associated with bullying (Landstedt and Persson, 2014). Generally, children and adolescents are the most vulnerable to being bullied (UNESCO, 2019), and the effects of bullying victimisation can last a lifetime (Takizawa *et al*., 2014; Hu, 2021). Moreover, bullying has a specific geographical distribution (Kim *et al*., 2022a, 2022b). The highest proportions of bullying victimisation among children and adolescents were found in Sub-Saharan Africa (48.2%), followed by North Africa (42.7%) and the Middle East (41.1%), and the lowest were found in Central America (22.8%), followed by Europe (25%) and the Caribbean (25%) (UNESCO, 2019).

Bullying has major links to anxiety disorders and major depressive disorder, with persistent impacts lasting through adulthood (Takizawa *et al*., 2014; Lereya *et al*., 2015; Islam *et al*., 2020; Ucar *et al*., 2020; Kim *et al*., 2022a, 2022b). Children with bullying experience were reported to have a 1.56 times higher risk of anxiety and a 1.80 times higher risk of depressive disorders (major depressive disorder and dysthymia) than their counterparts (Jadambaa *et al*., 2019). In a study of adult participants, those who were bullied in childhood had a 1.95 times higher odds of depression and a 1.65 times higher odds ratio of anxiety disorders at age 45 (Wolke *et al*., 2013). The possible mechanisms of the impact of bullying victimisation on mental disorders are as follows: (1) Based on emotional regulation theory, peer victimisation elicits strong negative emotions and may influence adolescents' ability to effectively manage emotional responses, which in turn impacts the development of internalising problems of anxiety and depression (Adrian *et al*., 2019); (2) Being bullied can affect adulthood through a stress process model in which bullying victimisation modifies stress responses or leads to long-term increases in inflammatory processes through traumatic stress that violence overwhelms children's psychological and biological stress process systems (Lereya *et al*., 2015), such as the hypothalamic–pituitary–adrenal axis (Arseneault *et al*., 2010).

Successful responses to address the challenge of bullying victimisation on mental disorders during childhood and adolescence require timely, reliable data at the national, regional and global levels to design effective interventions targeted at counteracting the disease burden generated by bullying victimisation. The characterisation of spatial and temporal trends as well as sociodemographic patterns of the burden of mental disorders attributed to bullying victimisation is crucial for identifying the potential protective mental health effects by public policies on bullying victimisation reduction. However, only a few studies have focused on the burden and trends of mental disorders attributable to bullying vision, and none of them have assessed differences across countries and regions of the world (Jadambaa *et al*., 2019; Yang *et al*., 2021). To fill this gap in knowledge, we used the Global Burden of Disease (GBD) dataset to analyse temporal and spatial trends in the bullying victimisation-related burden of anxiety disorders and major depressive disorder by sex, age, sociodemographic index (SDI) and country and territory. Our goal was to provide updated global evidence for public health policies in mitigating the burden of mental disorders attributed to bullying victimisation and developing prevention strategies tailored to different regions, countries and sociodemographic contexts.

## Methods

### Data sources

This study used secondary data from the 2019 GBD Study, which aimed to provide a comprehensive risk assessment approach of the disease burden of 369 diseases and injuries as well as comparative risks for 87 risk factors in 204 countries and territories from 1990 to 2019 (GBD 2019 Risk Factors Collaborators, 2020). In this study, the burden of disease attributable to bullying victimisation for different age groups (males, females and both sexes combined) and 204 countries and territories that were grouped into 21 regions was reported based on the comparative assessment approach. Further details of the general methodologies for GBD have been described elsewhere (GBD 2017 Risk Factor Collaborators, 2018); data and the protocol for the 2019 GBD can be accessed through the Global Health Data Exchange GBD Results Tool (http://ghdx.healthdata.org/gbd-results-tool). The study was compliant with the Guidelines for Accurate and Transparent Health Estimates Reporting.

### Definitions

#### Bullying victimisation

Bullying victimisation in the GBD context is defined as the 'bullying victimisation of children and adolescents attending school (aged 5–19 years) by peers', which incorporates combined estimates of subtypes such as physical, verbal, relational and cyberbullying victimisation. GBD 2019 incorporated data on bullying victimisation from 308 data sources (https://ghdx.healthdata.org/gbd-2019/data-input-sources). In the GBD analysis, two outcomes (anxiety disorders and major depressive disorder) for bullying were estimated. Further details have been provided elsewhere (GBD 2017 Risk Factor Collaborators, 2018).

#### Anxiety disorders and major depressive disorder

In the present study, we followed case definitions for anxiety disorders and major depressive disorder used by GBD, which were classified in accordance with the criteria presented in the Diagnostic and Statistical Manual of Mental Disorders (DSM-IV-TR) and the International Classification of Diseases and Related Health Problems (ICD-10) (World Health Organization, 1993; American Psychiatric Association, 2000; COVID-19 Mental Disorders Collaborators, 2021). In summary, the term anxiety disorders included generalised anxiety disorder, agoraphobia and panic disorder, social phobia, specific phobia and other unspecified anxiety disorders (Jadambaa *et al*., 2019). Anxiety disorders involve experiences of intense fear and distress, typically in combination with other physiological symptoms. Major depressive disorder involves the presence of at least one major depressive episode, which is the experience of either depressed mood or loss of interest or pleasure for most of every day, for at least 2 weeks. Further details are available elsewhere (GBD Collaborative Network, 2020a, 2020b).

#### DALYs

DALYs are defined as the sum of years lost due to premature death and years of healthy life lost due to disability (YLDs). DALYs were launched by the World Bank and are backed by the World Health Organization as a measure of the GBD (Arnesen and Nord, 1999). One DALY can be regarded as the loss of 1 year in full health (GBD Cancer Collaboration, 2019). More details on GBD 2019 modelling strategies for estimating cause-specific DALYs have been published elsewhere (GBD 2017 Disease and Injury Incidence and Prevalence Collaborators, 2018). To be more explicit, YLD was defined as ‘years lived with any short-term or long-term health loss weighted for severity by the disability weights' and YLD for a particular cause in a particular time period is estimated as follows: YLD = number of incident cases in that period × average duration of the disease × weight factor. The weight factor reflects the severity of the disease on a scale from 0 (perfect health) to 1 (death). More details on disability weight for anxiety disorders and major depressive disorder have been published elsewhere (Mathers *et al*., 2003; GBD Collaborative Network, 2012, 2016, 2017, 2018, 2020a, 2020b; Salomon *et al*., 2012, 2015) and an aggregate of disability weight could be found in online Supplementary Table S10.

#### SDI

The SDI used in this study was categorised into five groups: low-SDI (0–0.45), low-middle-SDI (0.45–0.61), middle-SDI (0.61–0.69), high-middle-SDI (0.69–0.81) and high-SDI (0.81–1) (Dai *et al*., 2020). SDI is a compound measure of income, average years of schooling and fertility for each GBD location and year, which was originally constructed for GBD 2015 using the Human Development Index (HDI) methodology to measure sociodemographic development.

#### Summary exposure value (SEV)

SEV was defined as a measure of exposure to a risk factor that takes into account the extent of exposure by risk level and the severity of that risk's contribution to disease burden. The metric is a risk-weighted prevalence of the population's exposure on a scale from 0 to 100%, where 0% reflects no risk exposure for a population and 100% indicates that the total population is at the highest level of that risk. All SEVs for each risk factor and how SEVs are computed could be found elsewhere (GBD 2017 Risk Factor Collaborators, 2018; GBD 2019 Risk Factors Collaborators, 2020).

### Analytic strategy

In this study, we used the modelling framework established for comparative risk assessment in GBD 2019 (GBD 2019 Risk Factors Collaborators, 2020) to examine the global DALYs of anxiety disorders and major depressive disorder attributable to bullying victimisation. We first used the percentage changes of the age-standardised DALY rates of anxiety disorders and major depressive disorder attributable to bullying victimisation to quantify the temporal trends by using DisMod-MR, a Bayesian meta-regression tool, and draws come from its posterior distribution with 95% uncertainty intervals (UIs), which were derived from the 25th and 975th values of the ordered 1000 draw-level estimates (GBD 2019 Diseases and Injuries Collaborators, 2020). The attributable proportions of age-standardised DALYs due to bullying victimisation were measured using population attributable fractions (PAFs), which were determined by the prevalence of exposure to bullying victimisation and the relative risks of disease occurrence. Additionally, we assessed the number of DALYs and age-standardised population rates (i.e. DALYs per 100 000 population) of the two outcomes attributable to bullying victimisation by age, sex and geographical location. The annualised rate of change by 204 countries and territories was used to present the spatial trends. Finally, we examined the relationship between SDI and the burden of anxiety disorders and major depressive disorder attributable to bullying victimisation and its spatial and temporal characteristics globally. Detailed information on analytical methods is available in previous studies (GBD 2017 Causes of Death Collaborators, 2018; GBD 2017 Disease and Injury Incidence and Prevalence Collaborators, 2018; GBD 2017 Risk Factor Collaborators, 2018). A 95% UI excluding 0 was considered to be statistically significant.

## Results

### Global exposure to bullying victimisation

Online Supplementary Table S1 shows global exposure to bullying victimisation. In 2019, the greatest risk exposure at the global level was in the 10–14 age group, with a SEV of 28.12 (95% UI 14.20–49.79), followed by the 15–19 age group, with a value of 22.64 (95% UI 9.94–45.25).

### Temporal trend of global burden of anxiety disorders and major depressive disorder attributable to bullying victimisation

In 2019, bullying victimisation caused 2.03 million (95% UI 0.59–4.42 million) DALYs of anxiety disorders globally, representing 7.47% (95% UI 2.31–15.15%) of anxiety disorder-related DALYs in both sexes combined (Table 1). Between 1990 and 2019, the age-standardised DALY rate of anxiety disorders attributable to bullying victimisation (per 100 000) increased from 21.84 (95% UI 6.21–48.42) to 26.93 (95% UI 8.02–58.28), a relative increase of 23.31% (95% UI 16.80–39.27) from the 1990 level in both sexes (Table 1).

**Table 1.** Global DALYs of anxiety disorders and major depressive disorder attributable to bullying victimisation for both sexes combined in 2019 and percentage change from 1990 to 2019 by GBD regions

	DALYs number	Age standardised PAF (%)	ASRs per 100 000	Percentage change in ASR, 1990–2019 (%)
Anxiety disorders				
Global	2 028 261.31 (587 420.84–4 422 058.83)	7.47 (2.31–15.15)	26.93 (8.02–58.28)	23.31 (16.80–39.27)
Andean Latin America	19 269.33 (4960.05–45 665.91)	5.44 (1.50–11.92)	28.75 (7.44–68.02)	12.12 (0.27–29.14)
Australasia	7776.69 (2085.86–17 885.71)	5.93 (1.84–12.64)	34.17 (9.86–76.09)	10.37 (−2.75 to 40.32)
Caribbean	11 865.32 (3126.57–28 116.61)	6.11 (1.76–13.11)	25.69 (6.90–59.98)	11.74 (5.27–22.13)
Central Asia	5334.00 (1315.62–13 176.60)	2.66 (0.71–6.02)	5.67 (1.46–13.82)	2.63 (−3.77 to 11.27)
Central Europe	15 001.89 (4031.42–35 227.46)	6.09 (1.93–12.43)	19.10 (5.61–42.53)	13.56 (0.62–44.67)
Central Latin America	62 858.57 (17 565.76–141 563.71)	6.39 (1.90–13.35)	24.07 (6.82–54.07)	40.76 (28.84–62.51)
Central Sub-Saharan Africa	48 058.88 (14 257.42–104 271.82)	8.14 (2.57–16.85)	29.91 (8.45–65.58)	36.45 (23.58–56.43)
East Asia	236 452.53 (68 223.15–517 807.88)	7.48 (2.57–14.76)	23.04 (7.31–47.94)	1.71 (−5.35 to 11.04)
Eastern Europe	39 648.12 (10 036.87–90 568.55)	8.77 (2.68–18.01)	26.74 (7.78–57.54)	19.29 (12.66–27.75)
Eastern Sub-Saharan Africa	122 320.68 (36 650.55–263 516.76)	6.76 (2.03–14.01)	23.96 (6.75–53.15)	50.92 (38.56–70.79)
High-income Asia Pacific	14 199.08 (3536.30–34 201.74)	4.99 (1.42–10.84)	12.63 (3.41–29.05)	2.15 (−3.60 to 10.21)
High-income North America	124 617.16 (28 143.49–291 362.03)	7.97 (2.01–17.49)	41.58 (9.95–95.73)	54.66 (39.68–74.65)
North Africa and Middle East	294 341.12 (95 969.42–622 992.40)	9.15 (3.43–17.20)	45.18 (14.95–94.92)	35.61 (16.46–78.61)
Oceania	3866.41 (1123.56–8636.70)	6.69 (2.08–14.18)	25.50 (7.30–57.86)	12.96 (2.24–28.35)
South Asia	505 214.92 (140 214.92–1 081 366.12)	8.61 (2.53–17.99)	24.68 (6.79–53.31)	25.69 (17.40–39.27)
Southeast Asia	137 863.35 (38 125.79–316 325.06)	5.78 (1.69–12.20)	20.12 (5.74–45.60)	36.37 (28.93–49.16)
Southern Latin America	24 678.14 (6584.20–53 938.78)	8.04 (2.42–16.92)	39.49 (11.16–84.52)	3.24 (−19.42 to 38.94)
Southern Sub-Saharan Africa	27 306.80 (7839.75–59 459.22)	9.11 (2.78–18.74)	31.52 (9.26–68.01)	11.99 (4.41–23.25)
Tropical Latin America	71 591.67 (16 926.36–176 956.24)	4.70 (1.24–10.43)	32.93 (8.45–79.81)	52.45 (37.52–69.42)
Western Europe	132 993.58 (38 208.93–296 781.56)	8.31 (2.80–16.90)	44.80 (13.86–97.25)	13.16 (−1.63 to 45.96)
Western Sub-Saharan Africa	123 003.08 (39 499.70–259 685.04)	7.38 (2.32–15.16)	21.71 (6.43–47.10)	49.96 (36.70–68.88)
Major depressive disorder				
Global	1 710 153.45 (353 088.57–3 995 310.23)	4.86 (1.19–10.09)	22.46 (4.77–52.18)	26.60 (18.14–47.22)
Andean Latin America	9095.23 (1868.34–21 494.27)	3.60 (0.89–7.78)	13.45 (2.80–31.92)	5.19 (−8.65 to 29.67)
Australasia	7426.37 (1605.43–17 604.01)	4.77 (1.24–10.08)	32.41 (7.57–73.75)	13.44 (−2.86 to 47.73)
Caribbean	10 019.82 (2127.34–24 203.36)	3.75 (0.93–8.09)	21.37 (4.64–51.10)	−8.21 (−16.23 to 3.56)
Central Asia	6346.78 (1094.69–16 143.73)	1.56 (0.34–3.59)	6.64 (1.22–16.51)	3.21 (−5.19 to 12.53)
Central Europe	8402.51 (1420.63–20 561.48)	3.39 (0.77–7.32)	10.38 (2.02–24.13)	−0.06 (−14.58 to 32.15)
Central Latin America	46 465.48 (9245.92–112 508.78)	3.64 (0.84–7.93)	17.50 (3.52–42.48)	44.28 (32.42–64.04)
Central Sub-Saharan Africa	62 878.76 (13 686.11–145 909.54)	4.72 (1.08–10.22)	41.19 (8.14–99.15)	29.43 (15.45–55.29)
East Asia	111 892.11 (18 793.27–286 771.07)	3.41 (0.80–7.24)	9.72 (1.98–23.12)	−25.37 (−31.69 to −14.60)
Eastern Europe	32 972.77 (4777.17–86 097.14)	4.65 (0.96–10.16)	21.20 (3.75–52.48)	14.75 (8.10–22.68)
Eastern Sub-Saharan Africa	119 675.84 (27 045.51–277 001.51)	3.40 (0.80–7.31)	24.09 (4.85–56.89)	42.17 (29.18–64.02)
High-income Asia Pacific	11 812.01 (2108.33–29 680.19)	3.24 (0.71–7.22)	9.94 (1.95–24.12)	19.56 (12.62–28.37)
High-income North America	162 567.07 (34 831.61–370 284.57)	8.53 (2.06–17.53)	54.81 (12.49–123.83)	105.88 (83.81–138.02)
North Africa and Middle East	243 080.91 (58 390.62–558 692.22)	5.47 (1.51–10.89)	36.97 (9.14–83.85)	37.34 (18.14–84.40)
Oceania	2540.21 (512.18–5959.77)	4.89 (1.14–10.70)	16.85 (3.31–40.05)	5.36 (−5.74 to 20.76)
South Asia	517 801.10 (103 668.52–1 203 667.55)	4.71 (1.06–9.92)	25.08 (4.92–59.45)	8.29 (−0.28 to 27.54)
Southeast Asia	79 541.03 (17 134.17–190 490.69)	4.49 (1.15–9.58)	11.50 (2.53–27.19)	23.38 (15.52–39.41)
Southern Latin America	17 626.38 (3542.88–40 892.20)	6.25 (1.47–13.16)	27.60 (5.94–62.95)	3.90 (−20.69 to 41.41)
Southern Sub-Saharan Africa	24 138.14 (4571.37–58 696.92)	4.75 (1.08–10.32)	27.53 (5.41–66.11)	13.03 (4.59–26.33)
Tropical Latin America	50 594.68 (11 267.11–119 963.89)	3.93 (1.03–8.27)	23.65 (5.56–55.28)	34.49 (17.36–68.06)
Western Europe	85 425.77 (16 846.35–201 213.20)	4.88 (1.17–10.30)	27.98 (6.13–64.33)	6.18 (−9.24 to 36.77)
Western Sub-Saharan Africa	99 850.48 (23 281.63–231 166.51)	3.30 (0.81–7.19)	18.64 (3.91–44.46)	40.92 (26.80–61.90)

Note: ASR, age-standardised rate; DALY, disability-adjusted life year; PAF, population attributable fraction; 95%UI, 95% uncertainty interval. A 95% UI excluding 0 was considered to be statistically significant.

Additionally, there was an estimated 1.71 million DALYs (95% UI 0.35–4.00 million) of major depressive disorder attributable to bullying victimisation in 2019, representing 4.86% (95% UI 1.19–10.09%) of major depressive disorder-related DALYs in both sexes combined (Table 1). The age-standardised DALY rate of major depressive disorder attributable to bullying victimisation in 2019 (22.46 per 100 000 [95% UI 4.77–52.18]) was 26.60% (95% UI 18.14–47.22%) higher than the 1990 level (17.74 per 100 000 [95% UI 3.47–43.11]). Overall, the numbers of DALYs and age-standardised rate (ASR) of anxiety disorders attributable to bullying victimisation were higher than those of major depressive disorder.

### Age and sex patterns of global burden of anxiety disorders and major depressive disorder attributable to bullying victimisation

**Fig. 1.**
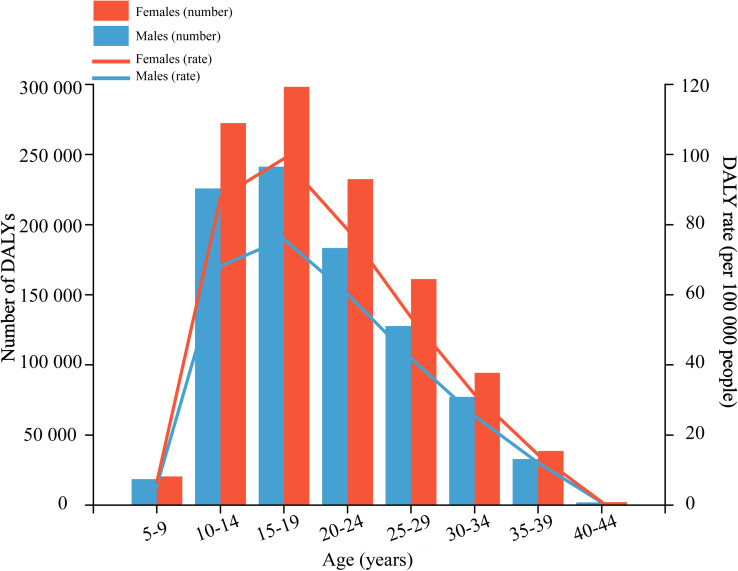
Age-specific numbers and rates of disability-adjusted life years (DALYs) of anxiety disorders attributable to bullying victimisation by sex, in 2019.

The age-specific DALY rates of anxiety disorders attributable to bullying victimisation increased with increasing age, peaking in the 15–19 age group, and then decreased; this pattern was similar for both males and females, with 0.24 million (95% UI 0.09–0.48 million) DALYs and 0.30 million (95% UI 0.10–0.61 million) DALYs, respectively (Fig. 1; online Supplementary Appendix Table 2). For males, bullying victimisation caused 0.91 million (95% UI 0.27–1.95 million) DALYs globally, representing 8.58% (95% UI 2.66–17.12%) of anxiety disorder-related DALYs and 1.12 million (95% UI 0.31–2.48 million) DALYs in females, with a PAF of 6.81% (95% UI 2.09–13.97%). Females had higher DALYs and ASRs of anxiety disorders attributable to bullying victimisation than males at all ages and a larger percentage increase could also be found in females from 1990 to 2019, increased by 27.27% (95% UI 19.62–44.46%) and 18.88% (95% UI 12.74–33.09%) for males (Table 2).

**Table 2.** Global DALYs of anxiety disorders and major depressive disorder attributable to bullying victimisation in 2019 and percentage change from 1990 to 2019 by sexes and GBD regions

	Male (95% UI)				Female (95% UI)			
	DALYs number	PAF (%)	ASRs per 100 000	Percentage change in ASR, 1990–2019 (%)	DALYs number	PAF (%)	ASRs per 100 000	Percentage change in ASR, 1990–2019(%)
Anxiety disorders								
Global	908 591.88 (270 055.95–1 945 681.32)	8.58 (2.66–17.12)	23.65 (7.20–50.44)	18.88 (12.74–33.09)	1 119 669.43 (314 546.92–2 480 222.68)	6.81 (2.09–13.97)	30.35 (8.79–66.59)	27.27 (19.62–44.46)
Andean Latin America	7815.96 (2088.52–18 382.46)	5.80 (1.62–12.81)	22.88 (6.14–53.81)	10.17 (−2.61 to 29.39)	11 453.36 (2838.31–27 315.20)	5.28 (1.45–11.55)	34.84 (8.76–82.56)	14.60 (0.66–34.68)
Australasia	3397.37 (924.54–7719.35)	6.65 (2.12–13.92)	29.62 (8.53–66.00)	12.70 (−3.62 to 43.06)	4379.31 (1157.48–10 176.07)	5.52 (1.66–11.85)	38.81 (11.02–87.20)	8.64 (−6.31 to 45.34)
Caribbean	5021.49 (1410.58–11 645.88)	6.94 (2.06–14.93)	21.70 (6.18–50.09)	10.29 (1.67–22.20)	6843.82 (1745.30–16 327.51)	5.66 (1.61–12.32)	29.70 (7.75–69.63)	13.24 (5.24–23.92)
Central Asia	2716.96 (674.40–6697.45)	3.48 (0.97–7.88)	5.66 (1.44–13.78)	0.96 (−7.31 to 11.82)	2617.04 (622.50–6562.82)	2.18 (0.55–5.06)	5.69 (1.38–14.05)	4.36 (−4.51 to 14.14)
Central Europe	6703.29 (1856.06–15 654.58)	7.32 (2.38–14.77)	16.62 (5.06–36.58)	13.35 (0.78–43.02)	8298.60 (2175.94–19 666.99)	5.44 (1.69–11.30)	21.72 (6.22–49.04)	13.94 (−0.03 to 49.74)
Central Latin America	23 940.95 (7044.41–53 671.43)	6.53 (1.98–13.62)	18.36 (5.42–41.19)	36.86 (23.47–63.78)	38 917.62 (10 510.69–88 531.17)	6.37 (1.88–13.67)	29.75 (8.25–67.44)	44.09 (30.24–64.79)
Central Sub-Saharan Africa	22 542.98 (6687.48–48 795.17)	9.13 (2.78–19.09)	28.45 (7.83–63.88)	23.79 (8.78–46.85)	25 515.90 (7593.23–54 932.78)	7.49 (2.38–15.45)	31.37 (8.90–68.96)	50.25 (30.99–79.59)
East Asia	107 510.82 (31 665.90–232 846.35)	8.26 (2.89–16.27)	19.82 (6.38–41.33)	0.00 (−6.84 to 10.54)	128 941.71 (36 418.89–285323.28)	7.03 (2.35–14.17)	26.68 (8.32–56.62)	4.06 (−4.94 to 15.57)
Eastern Europe	15 268.06 (3956.22–34 836.30)	8.80 (2.67–18.03)	20.20 (5.87–43.15)	27.59 (17.68–40.32)	24 380.05 (6211.41–55896.17)	8.99 (2.77–18.40)	33.56 (9.87–73.01)	15.05 (7.85–24.31)
Eastern Sub-Saharan Africa	54 221.39 (16 351.95–118 380.45)	7.44 (2.22–15.29)	21.55 (5.97–48.56)	44.31 (32.00–65.62)	68 099.30 (20 349.12–147333.87)	6.33 (1.93–13.14)	26.33 (7.30–57.75)	56.80 (42.42–80.40)
High-income Asia Pacific	7156.04 (1881.74–16 778.15)	6.36 (1.86–13.49)	12.43 (3.46–28.13)	1.78 (−6.34 to 11.61)	7043.04 (1626.99–17466.58)	4.10 (1.14–9.10)	12.84 (3.32–30.53)	2.62 (−5.92 to 13.74)
High-income North America	51 632.54 (11 953.19–119 572.34)	8.81 (2.25–18.64)	34.01 (8.42–76.44)	44.29 (27.02–62.61)	72 984.62 (16 259.65–173364.63)	7.54 (1.88–16.78)	49.40 (11.75–115.23)	63.16 (45.58–87.97)
North Africa and Middle East	157 637.74 (54 461.52–320 384.61)	12.52 (4.78–22.83)	46.80 (16.41–94.04)	16.98 (2.37–46.56)	136 703.39 (41 418.87–293329.38)	6.97 (2.39–13.56)	43.46 (13.40–93.33)	65.36 (35.39–142.41)
Oceania	1721.75 (505.14–3787.07)	7.50 (2.32–15.97)	21.88 (6.22–48.93)	11.75 (−2.76 to 31.10)	2144.66 (597.10–4930.41)	6.18 (1.91–13.18)	29.39 (8.13–68.18)	14.07 (0.36–34.57)
South Asia	225 996.20 (62 491.74–489 710.25)	9.14 (2.60–18.93)	21.52 (5.86–47.07)	14.49 (6.16–28.61)	279 218.72 (78 116.54–602558.45)	8.26 (2.46–17.34)	28.04 (7.81–60.51)	36.54 (23.75–58.11)
Southeast Asia	59 432.34 (16 822.97–135 092.47)	6.79 (2.00–14.26)	17.07 (4.98–38.34)	31.00 (22.56–45.26)	78 431.01 (21 352.93–182179.69)	5.26 (1.53–11.28)	23.28 (6.46–53.58)	41.29 (32.49–56.16)
Southern Latin America	9345.84 (2611.91–19 824.28)	9.27 (2.98–18.78)	29.95 (8.75–62.50)	12.46 (−12.04 to 51.23)	15 332.30 (4016.54–34278.82)	7.53 (2.16–16.13)	49.11 (13.74–107.19)	−1.31 (−25.49 to 35.83)
Southern Sub-Saharan Africa	12 614.78 (3648.85–27 149.77)	10.21 (3.12–20.63)	29.04 (8.58–62.08)	12.59 (3.20–25.95)	14 692.02 (4184.73–32212.84)	8.48 (2.61–17.59)	34.02 (9.87–74.02)	11.94 (3.08–23.96)
Tropical Latin America	27 169.15 (6520.77–64 110.56)	5.18 (1.40–11.48)	25.02 (6.28–57.89)	39.72 (24.38–59.77)	44 422.52 (10 227.91–109432.71)	4.50 (1.18–10.02)	40.89 (10.23–97.16)	62.07 (41.97–84.69)
Western Europe	52 568.70 (15 517.04–116 250.20)	9.25 (3.09–18.61)	34.53 (10.92–73.38)	14.72 (0.91–47.22)	80 424.89 (23 149.89–180279.85)	7.88 (2.67–15.96)	55.62 (17.07–122.64)	12.45 (−3.18 to 45.89)
Western Sub-Saharan Africa	54 177.53 (16 887.22–114 957.72)	7.82 (2.40–16.10)	19.99 (5.75–44.14)	37.13 (24.53–55.54)	68 825.55 (22 171.96–146950.59)	7.09 (2.28–14.55)	23.39 (7.10–50.72)	62.56 (44.41–90.38)
Major depressive disorder								
Global	751 822.67 (153 054.27–1 770 650.05)	5.45 (1.32–11.23)	19.37 (4.03–45.36)	23.84 (16.41–41.27)	958 330.78 (200 074.52–2245947.65)	4.53 (1.13–9.33)	25.68 (5.57–60.05)	29.07 (18.77–52.90)
Andean Latin America	3300.47 (685.65–7820.45)	3.62 (0.88–7.89)	9.56 (1.99–22.62)	8.26 (−7.27 to 37.34)	5794.76 (1191.70–13964.06)	3.65 (0.89–7.78)	17.51 (3.70–41.82)	5.20 (−10.31 to 31.50)
Australasia	2946.01 (624.44–7085.94)	4.66 (1.18–9.88)	25.31 (5.73–59.06)	−1.82 (−17.54 to 29.83)	4480.36 (963.16–10621.12)	4.88 (1.26–10.25)	39.73 (9.21–92.66)	26.33 (4.92–67.31)
Caribbean	3603.91 (737.30–8512.04)	3.81 (0.89–8.32)	15.31 (3.19–35.88)	−0.45 (−8.68 to 13.27)	6415.91 (1385.26–15599.23)	3.76 (0.96–8.13)	27.48 (6.07–66.33)	−11.35 (−20.84 to 1.02)
Central Asia	3405.55 (590.88–8609.81)	2.30 (0.50–5.20)	7.00 (1.29–17.34)	4.82 (−5.23 to 17.14)	2941.22 (484.43–7631.30)	1.18 (0.25–2.81)	6.27 (1.11–16.30)	1.28 (−8.83 to 11.29)
Central Europe	3653.54 (633.38–8933.44)	4.41 (1.00–9.44)	8.79 (1.74–20.60)	4.90 (−8.06 to 36.93)	4748.97 (802.72–11705.69)	2.96 (0.66–6.42)	12.06 (2.38–28.63)	−3.29 (−19.95 to 36.36)
Central Latin America	15 051.57 (2904.07–36 150.80)	3.38 (0.75–7.43)	11.39 (2.18–27.47)	35.59 (21.24–62.63)	31 413.90 (6285.10–76518.48)	3.84 (0.88–8.53)	23.58 (4.84–57.39)	50.06 (35.50–69.59)
Central Sub-Saharan Africa	29 531.07 (6418.43–68 446.55)	5.23 (1.17–11.29)	39.14 (7.55–93.73)	16.91 (2.98–42.57)	33 347.69 (7265.43–77823.55)	4.35 (1.01–9.58)	43.24 (8.71–102.94)	43.14 (22.08–83.26)
East Asia	47 955.73 (8171.72–120 481.42)	3.80 (0.88–8.11)	7.91 (1.60–18.87)	−19.19 (−26.24 to −7.86)	63 936.38 (10 450.69–165266.02)	3.23 (0.77–6.87)	11.74 (2.41–28.24)	−28.57 (−36.48 to −16.31)
Eastern Europe	14 152.03 (2060.27–37 217.04)	4.78 (0.99–10.45)	17.98 (3.15–44.55)	25.91 (15.55–38.40)	18 820.74 (2769.07–49344.10)	4.66 (0.96–10.20)	24.54 (4.30–60.53)	7.79 (0.41–15.94)
Eastern Sub-Saharan Africa	54 785.37 (12 119.39–125 766.15)	3.68 (0.86–7.96)	22.55 (4.46–53.15)	35.36 (22.77–57.58)	64 890.47 (14 791.66–149645.10)	3.21 (0.78–6.91)	25.61 (5.27–60.88)	48.60 (32.66–75.07)
High-income Asia Pacific	6467.98 (1233.50–16 041.00)	4.33 (1.01–9.40)	10.65 (2.28–25.28)	23.48 (13.16–37.31)	5344.03 (875.45–14 044.32)	2.50 (0.51–5.75)	9.19 (1.63–23.46)	14.98 (4.19–26.22)
High-income North America	58 915.49 (11 444.17–135 495.33)	8.52 (1.96–17.56)	38.67 (8.00–87.36)	78.00 (56.99–103.27)	103 651.58 (23 128.83–239 559.21)	8.61 (2.16–17.64)	71.61 (16.63–162.54)	125.74 (98.81–166.39)
North Africa and Middle East	127 799.05 (29 224.78–289 330.59)	7.17 (1.92–14.12)	37.41 (8.88–83.77)	20.52 (5.27–54.75)	115 281.86 (27 951.91–267 609.13)	4.33 (1.21–8.72)	36.47 (9.06–84.00)	61.43 (31.11–143.62)
Oceania	1308.96 (260.89–3098.68)	5.46 (1.20–12.14)	16.88 (3.24–40.66)	5.25 (−10.76 to 25.59)	1231.25 (253.75–2882.46)	4.41 (1.07–9.63)	16.88 (3.45–39.99)	5.60 (−8.60 to 26.41)
South Asia	230 497.11 (44 816.34–531 158.14)	5.00 (1.09–10.54)	21.83 (4.14–51.16)	1.74 (−7.04 to 18.92)	287 303.98 (57 363.66–668 343.35)	4.52 (1.02–9.63)	28.51 (5.68–66.62)	14.14 (1.57–41.21)
Southeast Asia	39 710.11 (8436.73–94 943.35)	5.17 (1.28–10.88)	11.29 (2.46–26.88)	19.29 (10.48–37.26)	39 830.92 (8449.99–94 553.40)	4.00 (1.02–8.56)	11.71 (2.55–27.73)	27.50 (18.26–43.95)
Southern Latin America	6511.33 (1288.69–14 885.26)	6.64 (1.48–14.22)	20.20 (4.24–45.70)	7.49 (−17.47 to 51.08)	11 115.05 (2245.46–26 229.91)	6.13 (1.47–12.90)	35.11 (7.64–80.58)	2.44 (−23.16 to 38.94)
Southern Sub-Saharan Africa	10 635.76 (2018.95–25 637.09)	5.51 (1.22–11.80)	24.25 (4.73–57.90)	22.06 (10.28–38.72)	13 502.38 (2580.28–32 582.06)	4.40 (1.00–9.66)	30.82 (6.13–73.53)	7.92 (−1.52 to 21.21)
Tropical Latin America	17 122.19 (3887.36–39 799.03)	4.20 (1.05–9.00)	15.82 (3.74–36.54)	36.52 (16.97–77.11)	33 472.49 (7366.50–80 480.13)	3.88 (1.03–8.18)	31.61 (7.38–74.34)	34.67 (15.71–71.35)
Western Europe	33 618.65 (6718.43–78 790.86)	5.28 (1.27–10.98)	21.42 (4.76–49.24)	2.76 (−11.02 to 32.51)	51 807.12 (10 275.32–121 973.60)	4.72 (1.13–9.98)	34.90 (7.61–80.31)	8.84 (−8.24 to 42.35)
Western Sub-Saharan Africa	40 850.78 (9530.77–93 273.26)	3.64 (0.87–7.83)	15.96 (3.33–37.84)	29.39 (16.80–50.22)	58 999.71 (13 620.58–136 627.34)	3.10 (0.77–6.78)	21.14 (4.46–50.13)	50.49 (31.39–84.23)

Note: ASR, age-standardised rate; DALY, disability-adjusted life year; PAF, population attributable fraction; 95%UI, 95% uncertainty interval. A 95% UI excluding 0 was considered to be statistically significant.

**Fig. 2.**
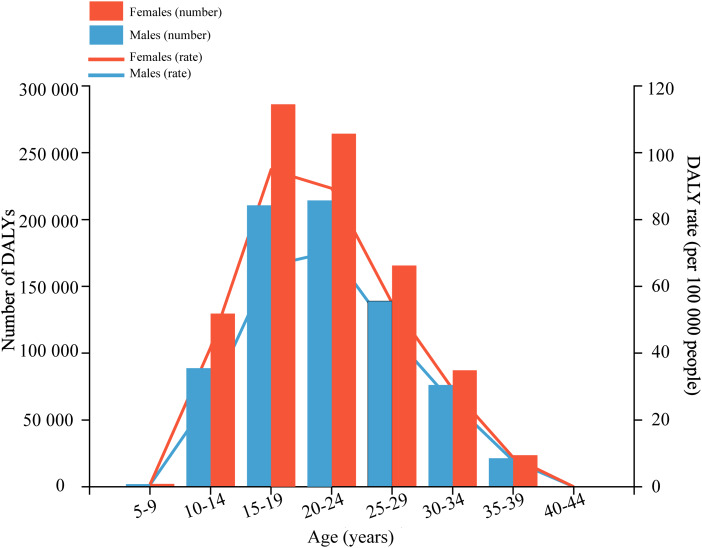
Age-specific numbers and rates of disability-adjusted life years (DALYs) of major depressive disorder attributable to bullying victimisation by sex, in 2019.

The age pattern for major depressive disorder was similar to that of anxiety disorders. Nevertheless, the DALY rate of major depressive disorder peaked in the age group of 20–24 years for males and 15–19 years for females, at 0.21 million (95% UI 0.04–0.48 million) DALYs and 0.29 million (95% UI 0.09–0.59 million) DALYs, respectively (Fig. 2; online Supplementary Appendix Table 2). Regarding the sex pattern for major depressive disorder, the global DALY rate attributable to bullying victimisation caused 0.75 million (95% UI 0.15–1.77 million) DALYs for males and 0.96 million (95% UI 0.20–2.25 million) for females, representing 5.45% (95% UI 1.32–11.23%) and 4.53% (95% UI 1.13–9.33%) of major depressive disorder-related DALYs, respectively. The number of DALYs and the ASR were also higher in females than in males of all ages, and so were the global age-standardised DALY rates with an increase of 29.07% (95% UI 18.77–52.90%) for females and 23.84% (95% UI 16.41–41.27%) for males from 1990 to 2019 (Table 2).

### Global burden of anxiety disorders and major depressive disorder attributable to bullying victimisation by 21 GBD regions and 204 countries and territories

Across 21 GBD regions, the top three age-standardised DALY rates of bullying victimisation (per 100 000) of anxiety disorders in 2019 were in North Africa and the Middle East (45.18 [95% UI 14.95–94.92]), Western Europe (44.80 [95% UI 13.86–97.25]) and High-income North America (41.58 [95% UI 9.95–95.73]). In contrast, the lowest three ASRs were observed in Central Asia (5.67 [95% UI 1.46–13.82]), High-income Asia Pacific (12.63 [95% UI 3.41–29.05]) and Central Europe (19.10 [95% UI 5.61–42.53]). All 21 GBD regions showed increases in the age-standardised DALY rate of anxiety disorders attributable to bullying victimisation from 1990 to 2019, with the largest increase being seen in High-income North America (54.66% [95% UI 39.68–74.65%]) and the lowest increase being found in East Asia (1.71% [95% UI −5.35 to 11.04%]) (Table 1). More details on DALYs and the PAF attributable to bullying victimisation can be found in Table 1.

**Fig. 3.**
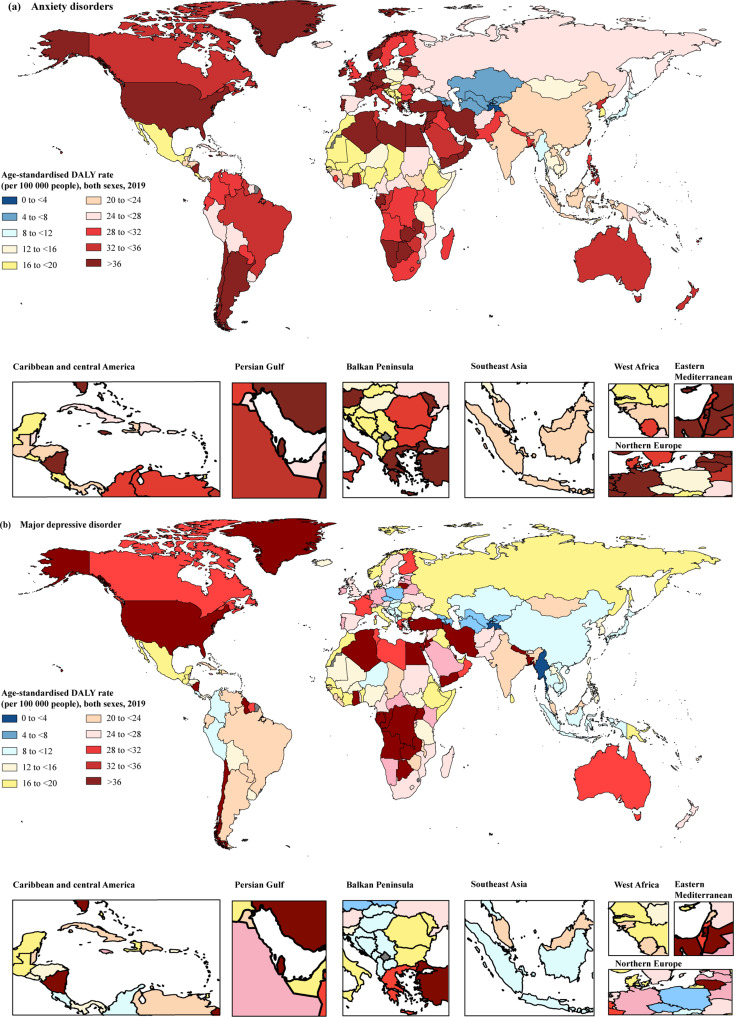
Age-standardised DALY rates of anxiety disorders (a) and major depressive disorder (b) attributable to bullying victimisation for both sexes in 2019. DALYs, disability-adjusted life years.

**Fig. 4.**
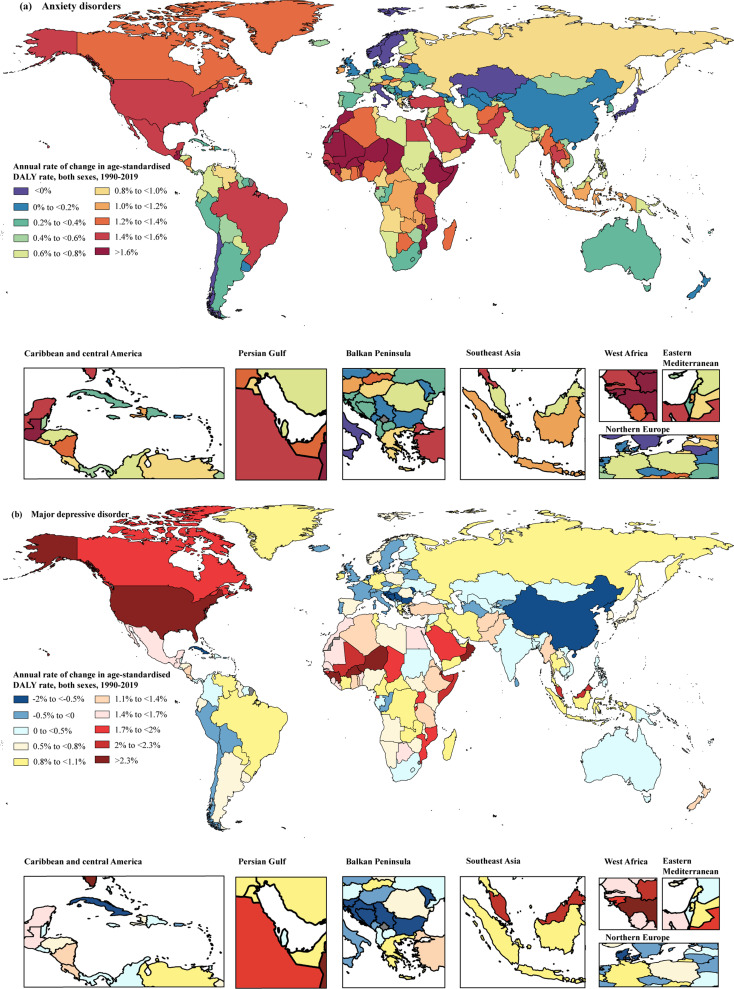
Annual rate change of age-standardised DALY rates of anxiety disorders (a) and major depressive disorder (b) attributable to bullying victimisation for both sexes from 1990 to 2019. DALYs, disability-adjusted life years.

Across 204 countries, in 2019, the countries with the top three age-standardised DALY rates of anxiety disorder attributable to bullying victimisation were Egypt, France and Austria, representing 16.46% (95% UI 6.71–28.66%), 10.79% (95% UI 3.71–20.33%) and 10.51% (95% UI 3.66–20.48%) of anxiety disorder-related DALY rates attributable to bullying victimisation, respectively, whereas the country with the lowest rates was Tajikistan, followed by Kyrgyzstan and Uzbekistan, representing only 1.42% (95% UI 0.37–3.44%), 2.71% (95% UI 0.70–6.30%) and 2.73% (95% UI 0.73–6.22%), respectively (Fig. 3a; online Supplementary Table S3). The highest annual rate change from 1990 to 2019 was in Niger (3.21%), whereas Taiwan (the Province of China) (−0.52%), Sweden (−0.38%) and Italy (−0.23%) were the three countries and territories that showed the largest declining annual rates of change from 1990 to 2019 (Fig. 4a). More information by country and sex on the percentage change in ASR from 1990 to 2019 is presented in online Supplementary Table S3, and the spatial trends using annual rate change can be found in Fig. 4a.

The age-standardised DALY rate of major depressive disorder attributable to bullying victimisation (per 100 000) was the highest in High-income North America (54.81 [95% UI 12.49–123.83]), followed by Central Sub-Saharan Africa (41.19 [95% UI 8.14–99.15]) and North Africa and the Middle East (36.97 [95% UI 9.14–83.85]). The lowest three rates were found in High-income Asia Pacific (3.24 [95% UI 0.71–7.22]), East Asia (3.41 [95% UI 0.80–7.24]) and Central Asia (1.56 [95% UI 0.34–3.59]) (Table 1). Most GBD regions (18 out of 21) showed percentage increases in the ASR of major depressive disorder attributable to bullying victimisation from 1990 to 2019, with the largest three percentage increase of ASR seen in High-income North America (105.88% [95% UI 83.81–138.02%]), Central Latin America (44.28% [95% UI 32.42–64.04%]) and Eastern Sub-Saharan Africa (42.17% [95% UI 29.18–64.02%]), whereas a negative decline was only seen in East Asia (−25.37% [95% UI −31.69 to −14.60%]), the Caribbean (−8.21% [95% UI −16.23 to 3.56%]) and Central Europe (−0.06% [95% UI −14.58 to 32.15%]). See Table 1.

At the national level, the age-standardised DALY rates of major depressive disorder attributable to bullying victimisation in 2019 ranged from 2.68 to 104.85 per 100 000 people. The countries or territories with the highest three age-standardised DALY rates of bullying victimisation (per 100 000 people) were Greenland (104.85 [95% UI 24.36–227.30]), Egypt (57.37 [95% UI 15.64–121.72]) and the United States (56.67 [95% UI 12.27–129.15]). The countries with the lowest ASRs per 100 000 for major depressive disorder were Tajikistan (2.68 [95% UI 0.49–6.81]), Myanmar (2.96 [95% UI 0.62–7.19]) and Armenia (4.46 [95% UI 0.84–11.07]) (Fig. 3b; online Supplementary Table S4). The annual rates of change in 163 out of 204 countries and territories increased from 1990 to 2019, with the highest annual rate of change in Niger (2.98%). In contrast, from 1990 to 2019, the annual rates of change in 41 countries and territories showed a downward trend, with Cuba (−1.33%), Bosnia and Herzegovina (−1.18%) and China (−1.04%) having the largest annual decline in DALY rates related to major depressive disorder attributed to bullying victimisation during this period (Fig. 4b).

### Relationship between SDI and the impact of bullying victimisation on the global burden of anxiety disorders and major depressive disorder

**Fig. 5.**
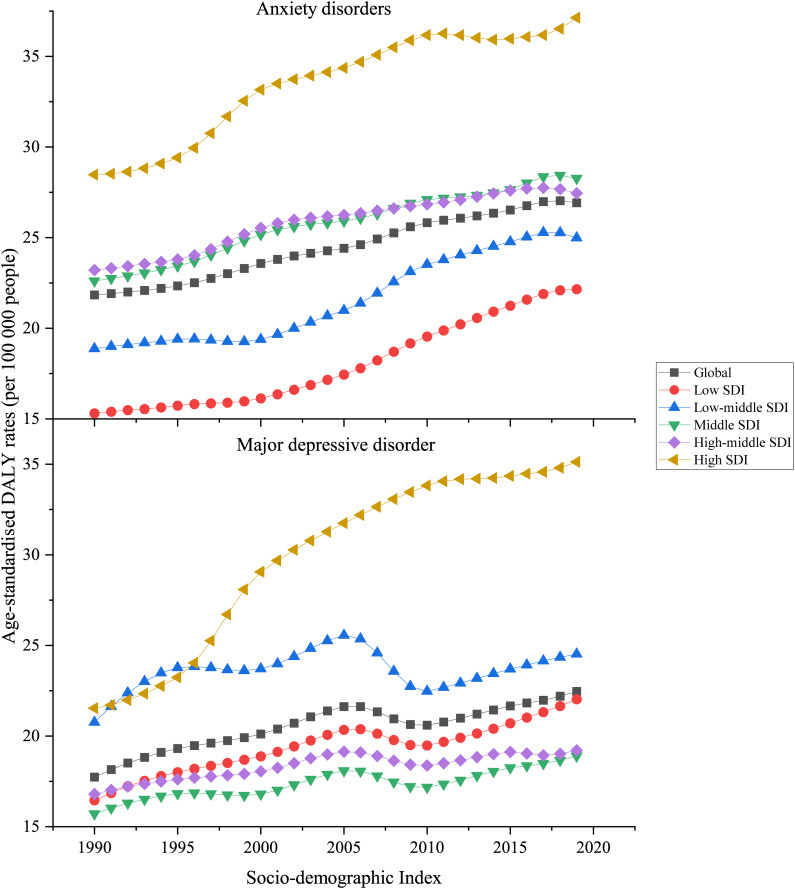
Age-standardised DALY rates attributable to bullying victimisation in five SDI groups for both sexes combined, 1990–2019. All countries and territories were categorised according to sociodemographic index quintile into five groups: low-SDI, low-middle-SDI, middle-SDI, high-middle-SDI and high-SDI quintile.

There was generally a positive association between regional SDI and the corresponding age-standardised DALY rates of anxiety disorders attributable to bullying victimisation from 1990 to 2019 (Fig. 5). As SDI increased, the age-standardised DALY rate increased; although, the magnitude of age-standardised DALY rates of anxiety disorders differed by SDI levels. High SDI quintile countries had the highest age-standardised DALY rates, whereas low SDI quintile countries had the smallest DALY rates. In 2019, the anxiety disorder-related DALY rate of the high SDI quintile area was 37.14 (95% UI 10.88–81.80) DALYs per 100 000 people, and the rate of the low SDI quintile area was 22.17 (95% UI 6.29–48.98) DALYs per 100 000 people (Fig. 5).

**Fig. 6.**
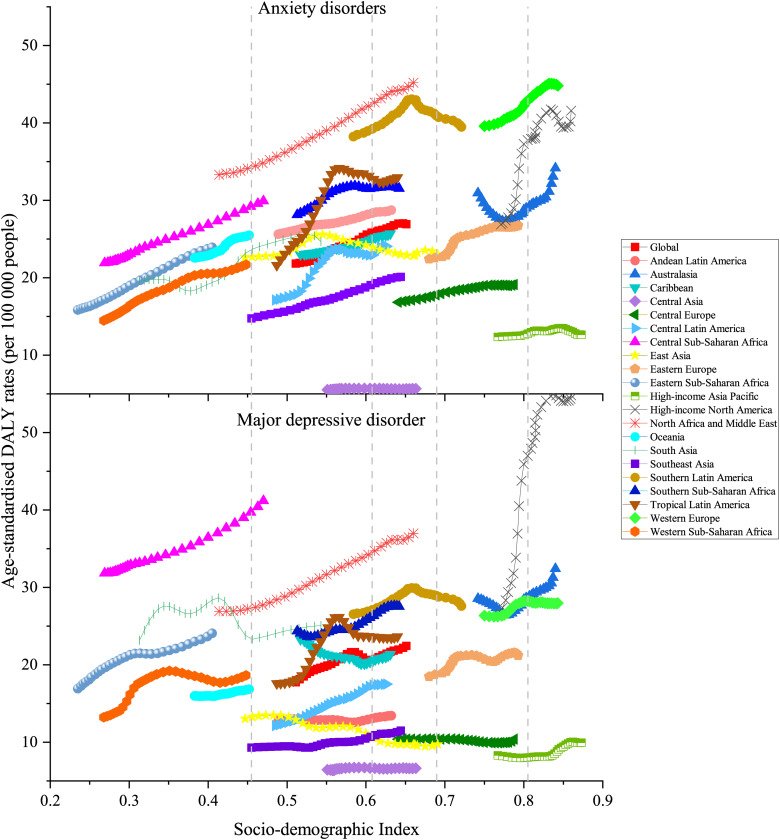
Age-standardised DALY rates of anxiety disorders and major depressive disorder attributable to bullying victimisation across 21 GBD regions by sociodemographic index for both sexes combined, 1990–2019.

The changes in anxiety disorder-related age-standardised DALY rates attributable to bullying victimisation across SDI by 21 GBD regions can be found in Fig. 6. Among all five GBD regions with the lowest SDI, four of them (Central, Eastern and Western Sub-Sahara Africa areas and Oceania) experienced increasing rates. Additionally, there were four different patterns in low-middle SDI quintile countries, including an inverted-U-curve in Tropical Latin America and Central Latin America declining until 0.56 SDI, a stable pattern in Central Asia and Andean Latin America, and an increasing growth pattern in the Caribbean, South-East Asia, and North Africa and the Middle East. Of the regions with middle SDI and high-middle SDI, two regions exhibited inverted-U-curves in Southern Latin America and Eastern Europe, while one region (Central Europe) remained stable. Of the four GBD regions with the highest SDI, three (Western Europe, Asia Pacific and High-income North America) exhibited an inverted-U-curve, one (Australasia) experienced a growth pattern.

For the relationship between SDI and the burden of major depressive disorder attributed to bullying victimisation, the largest value of age-standardised DALY rate of major depressive disorder was found in high SDI quintile countries, with 35.12 (95% UI 7.94–78.65) DALYs per 100 000 people in 2019. The smallest value of major depressive disorder DALYs rate was in countries in the middle SDI quintile, with 18.89 (95% UI 4.21–44.39) DALYs per 100 000 people in 2019 (Fig. 5).

In low SDI quintile countries, there was an increase in the age-standardised DALY rate of major depressive disorder attributed to bullying victimisation in Central Sub-Saharan Africa, Eastern Sub-Saharan Africa and Oceania. In countries with a low-middle SDI, there are several different patterns, including an inverted-U-curve in Tropical Latin America declining until 0.56 SDI, an increasing growth pattern in North Africa and the Middle East, Southern Sub-Saharan Africa, Southeast Asia and Central Latin America. Of the regions with the lowest rates in countries with middle SDI quintiles, East Asia showed a decrease in rates, Central Europe and Central Asia remained stable, and Southern Latin America exhibited an inverted-U-curve. Of the four GBD regions with the highest SDI quintile, one exhibited an inverted-U-curve with an increase first and then a decline in Western Europe, whereas the rates in Asia Pacific and North America experienced growth but remained stable when the SDI reached approximately 0.85.

## Discussion

Using the available data and analytical framework of GBD 2019, this study contributed to existing literature on GBD by investigating the temporal and spatial trends in burdens of anxiety disorders and major depressive disorder attributable to bullying victimisation from 1990 to 2019. Our findings showed that the age-standardised DALY rates for anxiety disorders and major depressive disorder attributable to bullying victimisation increased by 23.31 and 26.60%, respectively. One possible reason driving this increase is a concurrent increase in bullying prevalence (UNESCO, 2019).

Our findings showed that sex differences existed in the burden of anxiety disorders and major depressive disorder attributable to bullying victimisation. Although boys were exposed to a higher proportion of bullying victimisation than girls, higher age-standardised DALY rates of mental disorders were observed in girls. Two possible reasons for girls suffering more psychosomatic problems associated with bullying are as follows: First, sex differences exist in the response to stressors (e.g. peer victimisation), which may contribute to sex heterogeneity in the effects of bullying victimisation on mental disorders. Previous evidence suggested that girls with more passive reactions to traumatic events and who are more likely to internalise stress tended to have higher risks of anxiety and depressive symptoms, in comparison with boys (Chang *et al*., 2019; Vasiliadis *et al*., 2020; Roza *et al*., 2021). Second, sex differences in types of bullying exposure may be the other reason for our findings. Compared with boys experiencing physical bullying, girls tend to experience psychological bullying, cyberbullying via messages and bullying based on their physical appearance (UNESCO, 2019), which subsequently leads to unhealthy psychological conditions (He *et al*., 2022).

Substantial geographical variations were found in bullying victimisation related to mental disorders burden. For example, High-income North America, Central Sub-Saharan Africa, and North Africa and the Middle East were the top three regions with DALY rates of major depressive disorder related to bullying victimisation, whereas High-income Asia Pacific, East Asia and Central Asia were the lowest three regions. Regional differences in cultural diversity, implementation of policies on anti-bullying, types of bullying which victims have been exposed to are possible reasons for these regional differences.

First, cultural diversity-related factors, such as a higher proportion of immigrants and refugees or gender-nonconforming populations, may lead to conflicts occurring between individuals or social groups separated by cultural boundaries, leading to high-risk factors of bullying-related mental disorders in places like High-income North America. For instance, in the United States with a large population of immigrants and refugees, as compared to native-born youth, immigrant youth are at high risk for experiencing bullying victimisation and report more interpersonal, socioemotional, health and substance use problems (Maynard *et al*., 2016; UNESCO, 2019). As compared to students who identified as heterosexual, students who identified as lesbian, gay, bisexual, transgender (LGBT) or unsure about their sexual orientation are significantly more likely to be bullied due to bullying towards sexual and gender minorities, which may be due to predominant norms of what are acceptable practices of sexuality (Kann *et al*., 2016; Lowry *et al*., 2020; McCormick and Krieger, 2020). To prevent bullying victimisation, societies should understand how bullying-supportive norms are located and why the public accept and even reinforce these norms. Second, children and adolescents in High-income North America, compared with those in East Asia and Central Asia, may be exposed to more types of bullying in addition to traditional bullying, such as new forms like cyberbullying. Children and adolescents in High-income North America have a greater access to electronic devices and may have greater exposure to the online environment (Bannink *et al*., 2014), which leads to cyberbullying and greater burden of bullying victimisation.

Four temporal and spatial patterns of bullying victimisation-related mental disorders were found: (1) increasing patterns were prominent in Africa, the Middle East, Australasia and High-income North America; (2) decreasing patterns were the most common in East Asian areas; (3) inverted-U-curve patterns were found in areas of Tropical Latin America, Southern Latin America and Western Europe; and (4) most of the areas with stable patterns were concentrated in southeast Asia and the High-income Asia Pacific. The anti-bullying policies and culture-related factors may partially explain these differences in patterns:

First, the implementation of national-wide anti-bullying policies may be important contributors of the decline and inverted-U-curve patterns. One such case can be seen in Western Europe. For example, the implementation of anti-bullying policies in Finland in 2009, and Sweden in 2010 (Hatzenbuehler *et al*., 2015; Arseneault, 2018), as well as anti-bullying programmes such as KiVa and NoTrap! developed in Finland, the Netherlands and Italy (Huitsing *et al*., 2020; Zambuto *et al*., 2020), may have delayed effects leading to decreases in the burden of bullying-related anxiety disorders, with a turning point after 2017. Second, regarding culture-related factors, the Confucian tradition and collectivistic culture in East Asia, which emphasise discipline and has less tolerance of bullying (Jin *et al*., 2019), may contribute to stable low temporal trends of bullying-related mental disorders in the region. Thereby, schools need to purposively create an ethos of gender equality and respectful relationships in order to have a positive impact on students' attitude and behaviours. However, mental disorders may also be underreported in East Asia and other regions due to cultural stigma and differences in how mental health is experienced (Bharadwaj *et al*., 2017; Ken-Opurum *et al*., 2020). In addition, regions with increasing patterns may be experiencing cultural conflicts, such as immigration and gender-nonconforming as mentioned above. Besides, in High-income Australasia, this situation may also result from culturally and linguistically diverse backgrounds (Australian Institute of Health Welfare, 2022), especially for Indigenous youth due to the historical colonisation and assimilative policies (Islam *et al*., 2022) while in Arab society, the serious situation might be amplified by gender restrictive norms that girls and women are required to conform to traditional roles by submitting to men and remain silent after experiencing sexual victimisation (Jeries-Loulou and Khoury-Kassabri, 2022). Bullying victimisation driven by cultural conflicts may not be entirely addressed by anti-bullying policies. For example, in the United States, even with more than 120 bills related to bullying passed between 1999 and 2010 (Hatzenbuehler *et al*., 2015), although short decline for disease burden of both anxiety disorders and major depressive disorder was observed around 2012, the increase in bullying starting around 2016 may be influenced by a variety of factors, such as increasing diversity of students in terms of race and sexual orientation and the reluctance of schools in taking responsibility for anti-bullying policy implementation (Chen *et al*., 2021).

Regarding SDI, this study found there were overall upward trends of bullying-related mental disorders in areas regardless of SDI levels, although there were transient downward trends in certain areas. Interventions to prevent bullying victimisation and promote the mental health for victims are crucial to mitigate these increasing trends. Therefore, it is crucial to consider factors that increase the risk of bullying victimisation (e.g. lax implementation of policy, social inequality, gender norms, culture of violence, cultural conflicts) and to increase those that protect from bullying victimisation (e.g. children rights' empowerment, parenting support, school-based supportive team) and successive interventions are needed to prevent bullying victimisation and promote mental health for victims. In addition to advocating for supportive legal and policy frameworks, reducing cultural conflicts through creating safe and inclusive school-based and home-based environments (Hatzenbuehler *et al*., 2015), and reaffirming strong commitments to children's rights and empowerment are considered effective measures (Zambuto *et al*., 2020). As for children and adolescents who have been bullied, peer approaches to involve all students and motivate bystanders to stand up for victims are also proven methods for reducing bullying-related mental disorders (Huitsing *et al*., 2020). Other methods that may reduce the negative impact on mental health caused by bullying include providing school-based support (such as referral to support teams comprised of social workers, social educators and psychologists who can provide access to trained counsellors); offering care and access to justice; and teaching coping skills to strengthen students' social and emotional skills for dealing with stressful situations and improving emotional regulation (Arseneault, 2018; UNESCO, 2019).

To the best of our knowledge, this study is the first to analyse temporal and spatial trends in bullying victimisation-related burden of anxiety disorders and major depressive disorder. We used the most recent GBD 2019 data to examine trends on global, regional and country scales covering a span of 30 years. However, some limitations still exist. First, bullying victimisation in general has been reported here, so we were unable to investigate the attributable burdens of specific types of bullying, such as physical, psychological and cyberbullying. Future studies that include traditional and new forms of bullying are needed to address this critical research question. Second, since some estimates of the disease burden continued to rely on sparse datasets and high-quality survey data remain scarce for many countries, the result may be an underestimate, especially in some underdeveloped regions, such as Africa and South Asia (Yang *et al*., 2021; GBD 2019 Mental Disorders Collaborators, 2022). This might be explained by several reasons. On the one hand, the recognition and diagnosis rate of certain diseases is lower in developing countries due to the lower coverage of epidemiological surveys (GBD 2019 Mental Disorders Collaborators, 2022). On the other hand, the bullying victimisation reporting rate might be underestimated because universal primary and secondary education is not mandatory, and school-based surveys may not reach children who have already dropped out of school (Ruscio *et al*., 2017; UNESCO, 2019). Furthermore, even when data are available, they might not have been obtained using the preferred case definition or measurement method (GBD 2019 Diseases and Injuries Collaborators, 2020). Moreover, self-report bias is also one potential limitation, as bullying victimisation measurements might differ between regions with respect to different cultures and backgrounds, and data collection methods might be non-uniform across geography.

## Conclusions

The global burden of anxiety disorders and major depressive disorder attributable to bullying victimisation continues to increase over the past 30 years, with marked variations by sex and geographical region. Effective strategies, such as establishing a supportive legal and policy framework, relieving cultural conflicts, such as creating a safer and inclusive school-based and home-based environments, increasing access to specialists of bullying victimisation-related mental disorder services, and teaching coping skills to strengthen students' social and emotional skills may be useful for reducing the burden of anxiety disorders and major depressive disorder. Given the large variations in the burden by sex, SDI and geographic location, future actions should be designed and implemented based on the specific development status, as well as cultural and regional characteristics of each country.

## Data Availability

Data are available in the Global Health Data Exchange GBD Results Tool, http://ghdx.healthdata.org/gbd-results-tool.

## Supplementary material

For supplementary material accompanying this paper visit http://doi.org/10.1017/S2045796022000683. click here to view supplementary material
